# Attenuation of Olanzapine-Induced Endoplasmic Reticulum Stress Improves Insulin Secretion in Pancreatic Beta Cells

**DOI:** 10.3390/metabo12050443

**Published:** 2022-05-16

**Authors:** Diana Grajales, Patricia Vázquez, Rosa Alén, Ana B. Hitos, Ángela M. Valverde

**Affiliations:** 1 Instituto de Investigaciones Biomédicas Alberto Sols, Consejo Superior de Investigaciones Científicas (CSIC), 28029 Madrid, Spain; dgrajales@iib.uam.es (D.G.); patrivazquez@iib.uam.es (P.V.); ralen@iib.uam.es (R.A.); ahitos@iib.uam.es (A.B.H.); 2 CIBER de Diabetes y Enfermedades Metabólicas Asociadas (CIBERDEM), Instituto de Salud Carlos III, 28029 Madrid, Spain

**Keywords:** olanzapine, ER stress, beta cell, second-generation antipsychotics, schizophrenia, type 2 diabetes

## Abstract

Second-generation antipsychotics (SGAs), in particular, olanzapine and clozapine, have been associated with the development of type 2 diabetes mellitus (T2D) and metabolic syndrome in individuals with schizophrenia. In this context, beta cell dysfunction is a plausible mechanism by which SGAs cause T2D. Herein, we analyzed the direct effects of olanzapine, a commonly prescribed SGA with diabetogenic properties, on the INS-1 (821/13) beta cell line and isolated pancreatic islets. Treatment of INS-1 beta cells with non-toxic concentrations of olanzapine (3–6 μM) during 4 h activated endoplasmic reticulum (ER) stress-mediated signaling by increasing PERK/eIF2α phosphorylation, IRE-1 phosphorylation and XBP-1 splicing. Moreover, glucose-stimulated insulin secretion (GSIS) was inhibited when olanzapine was present for 16 h. The insulin secretory function of INS-1 cells was restored by inhibiting olanzapine-induced ER stress with tauroursodeoxycholic acid (TUDCA). Similar effects of olanzapine with or without TUDCA on ER-stress-mediated signaling and GSIS were found in pancreatic islets from female mice. Our results indicate that early activation of ER stress in pancreatic beta cells is a potential mechanism behind the alterations in glucose homeostasis induced by olanzapine.

## 1. Introduction

Second-generation antipsychotics (SGAs) are the main line of treatment for schizophrenia, and other severe mental illness (SMI), due to their higher clinical efficacy compared to first-generation antipsychotics (FGA) [1]. However, patients prescribed SGAs experience a plethora of metabolic side-effects, including weight gain, hyperglycemia, dyslipidemia and insulin resistance, which can lead to type 2 diabetes (T2D). Since weight gain is directly related with insulin resistance, hyperinsulinemia and T2D [2], research on the molecular basis of the obesogenic effect and related metabolic alterations induced by SGAs is currently of high interest [3].

The different SGAs vary in their relative diabetogenic effects. In this regard, clozapine and olanzapine induce the greatest weight gain and confer a higher risk for metabolic disturbances compared with other antipsychotics [4]. However, the tremendous complexity of SGA pharmacology as antagonists or agonists of multiple dopamine, serotonin or cholinergic pathways, makes it challenging to determine the specific molecular alterations driven by these drugs in multiple organs. Broadly stated, SGAs’ effects appear to be mediated by interference in both the central nervous system (CNS) and the periphery, including metabolic tissues such as liver, adipose and pancreas [5].

Despite numerous investigations, controversy remains over the effects of SGAs on insulin secretion and beta cell function, effects that seem to be dependent on the antipsychotic binding to different receptors, and on the dose and treatment time [5]. In this regard, pancreatic beta cells express dopaminergic (D1R–D2R), serotonergic (5-HT) and muscarinic (M3R) receptors, D2R and MR3 antagonism being tightly linked to insulin secretion and beta cell function, as we recently reviewed [6]. Olanzapine has higher antagonistic affinity for serotonin 5-HT2A and dopamine D2R receptors. Additionally, it has antagonistic activity toward dopamine D3R and D4R, serotonin 5-HT3 and 5-HT6, histamine H1, α1-adrenergic receptor and muscarinic M1R-M5R [7].

Beta cells are extremely dependent on the endoplasmic reticulum (ER) to sustain insulin production. In situations of insulin resistance, beta cells increase the production of insulin to compensate for the higher peripheral insulin demand, thereby enhancing ER function. However, exceeding the functional capacity of the ER can lead to accumulation of misfolded proteins and activation of the unfolded protein response (UPR) [8]. The UPR is an adaptive response aimed to improve the ER’s capacity to fold proteins, but excessive overload can lead to chronic ER stress activation and apoptosis, causing beta cell dysfunction [9]. In mammalian cells, the UPR signaling pathway is mediated by three ER membrane stress sensors: inositol requiring kinase 1α (IRE1α), activating transcription factor 6 (ATF6) and protein kinase RNA-like ER kinase (PERK). Among the UPR branches, PERK/eIF2α plays a major role in beta cell failure, where mutations have been related to the development of diabetes [10].

Previous studies have illustrated that the most diabetogenic SGAs, including olanzapine, clozapine and risperidone, induce ER stress in pancreatic beta cells [11], liver [12,13] and hypothalamus and prefrontal cortex [14,15]. An early study reported that treatment of the immortalized hamster pancreatic beta cell line HIT-T15 with 100 μM olanzapine, a concentration that significantly increased the number of apoptotic cells, induced ER stress via PERK phosphorylation, but independently of its downstream effector eIF2α [11]. In a recent study conducted on the mouse pancreatic beta cell line MIN6, olanzapine used at 10–50 μM reduced the maturation of proinsulin, and as a consequence, insulin secretion [16]. In the present study, we evaluated the effects of lower concentrations of olanzapine than those used in the above-mentioned studies on the INS-1 beta cell line and pancreatic islets, in order to avoid the subsequent effects of this SGA in insulin secretion related to cell death. Data showed that olanzapine activated PERK/eIF2α- and IRE1α -mediated signaling and decreased insulin secretion. Of relevance, the functionality of INS-1 beta cells and isolated islets was restored by inhibiting ER stress with tauroursodeoxycholic acid (TUDCA). Therefore, targeting ER stress in pancreatic beta cells may have potential translational value by preventing metabolic alterations in individuals under chronic treatment with olanzapine.

## 2. Results

### 2.1. Treatment of INS-1 Beta Cells with Olanzapine Reduces Cell Viability in a Dose-Dependent Manner

**Figure 1:**
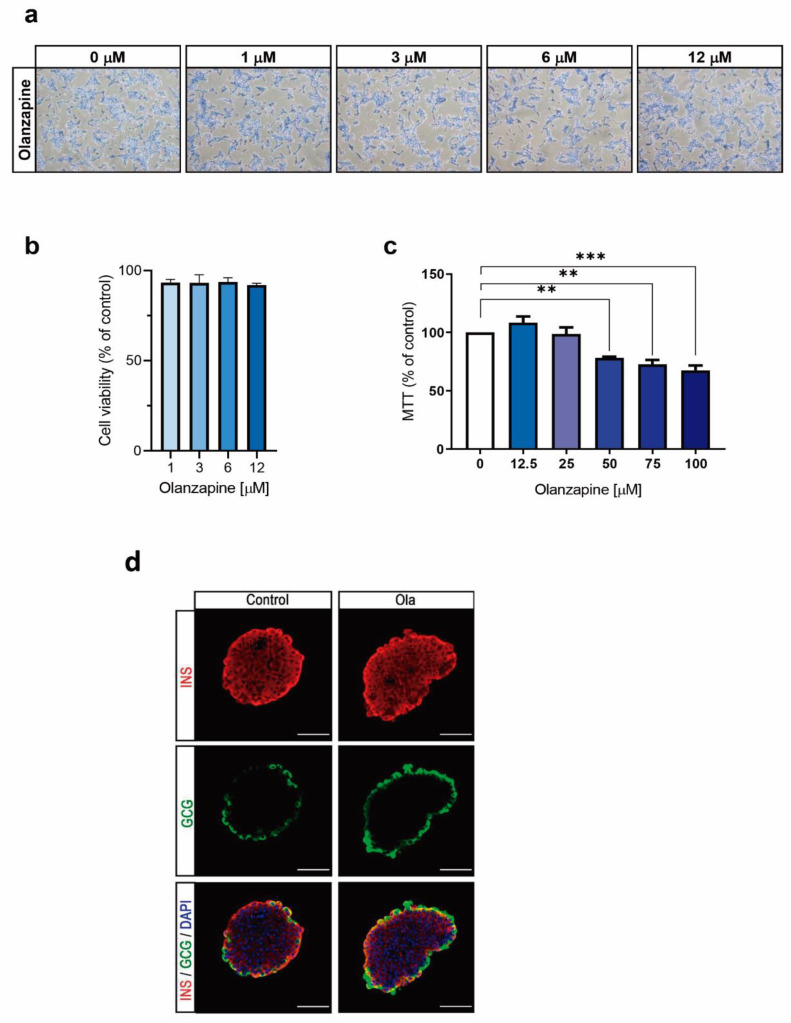
Effect of olanzapine on the cellular viability of INS-1 cells after 24 h of treatment. (**a**) Representative crystal violet staining images captured with a light microscope at 10× magnification. (**b**) Cell viability monitored by crystal violet assay, represented as the percentages of viability compared to control cells. (**c**) Cell viability measured by the MTT assay relative to control cells. Data are shown as percentages of viability compared to control cells. Control cells were treated with DMSO (0.01%). (**d**) Representative images of confocal immunofluorescence of islets upon treatment with 6 μM olanzapine for 24 h. Insulin (in red) and glucagon (in green). 40× magnification. Scale bar = 50 μm. Experiments were performed in 20 islets per condition. Data are presented as means ± SEM, *n* = 3 independent experiments (3 replicates/condition), passage 25–31. *p*-values were determined by two-way ANOVA and Bonferroni post-hoc test. ** *p* < 0.01, *** *p* < 0.001, comparison between treatments.

Based on the concentrations used in previously reported in vitro studies with olanzapine in beta cells lines [11,17], we evaluated the effects of lower concentrations of this SGA (1, 3, 6 and 12 μM) on the viability of INS-1 cells. As shown in Figure 1a,b, treatment for 24 h with increasing concentrations of olanzapine of up to 12 μM did not reduce cell viability in INS-1 cells. However, the MTT cell viability assay revealed a significant reduction in olanzapine-treated INS-1 beta cells at concentrations above 50 μM (Figure 1c). We also tested the possible deleterious effects of olanzapine in the architecture of the pancreatic islets isolated from female mice, and as shown in Figure 1d, islets exposed to 6 μM olanzapine for 24 h showed a similar structure to non-treated islets without alterations in alpha or beta cell distribution.

### 2.2. Short-Term Treatment with Olanzapine Induces Endoplasmic Reticulum (ER) Stress-Mediated Signaling in INS-1 Cells and Pancreatic Islets

**Figure 2:**
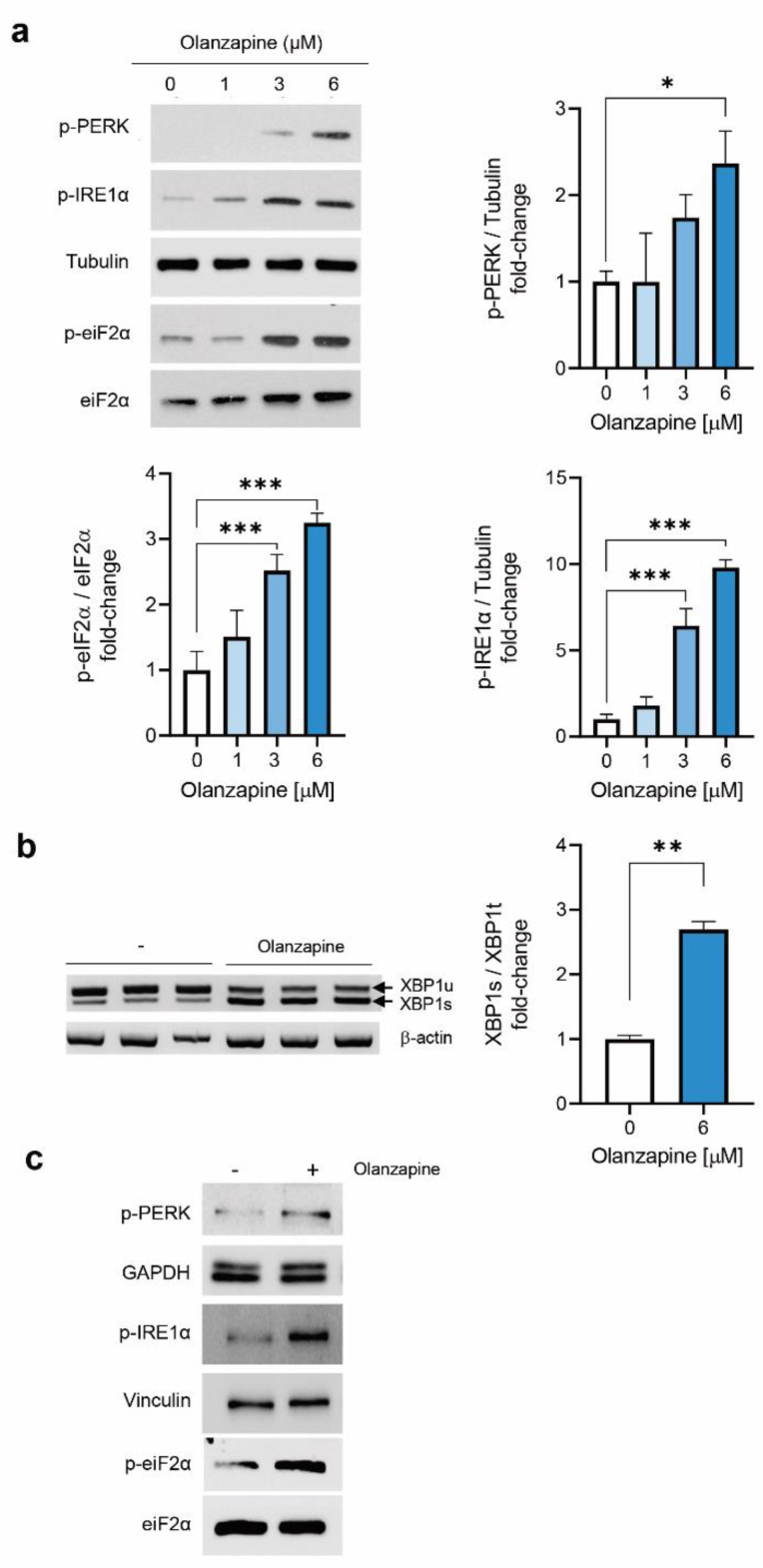
Effects of olanzapine treatment on ER-stress-mediated signaling in INS-1 beta cells and pancreatic islets. (**a**) Western blot analysis of the effect of olanzapine in ER stress markers in INS-1 beta cells after 4 h of treatment showing phosphorylation levels of PERK, eIF2α and IRE1α. Control: 0.01% DMSO was used as a vehicle. Representative Western blot images are shown. P-PERK, p-IRE1α and tubulin correspond to the same gel, and p-eiF2α and eiF2α correspond to the same gel. The graphs show the fold increases versus control cells. Tubulin and total eIF2α were used as loading controls. The experiments were repeated six times, independently, with INS-1 cells in different passages, with triplicate conditions in each one. Data are presented as mean ± SEM of *n* = 6 independent experiments (3 replicates/condition), passage 25–35. *p*-values were determined by one-way ANOVA and Bonferroni Post-hoc test. * *p* < 0.05, *** *p* < 0.001 versus control (non-treated) cells. (**b**) XBP1 splicing analyzed in INS-1 beta cells treated with olanzapine under similar experimental conditions. Data are presented as mean ± SEM of *n* = 2 independent experiments (3 replicates/condition). Differences between 2 groups were compared using the Mann–Whitney U test ** *p* < 0.01 versus control (non-treated) cells. (**c**) Pancreatic islets from female mice were incubated with 6 μM olanzapine for 4 h, and phosphorylation levels of PERK, IRE1α and eIF2α were analyzed by Western blot. Representative Western blot images from 2 independent experiments are shown. P-PERK and GAPDH correspond to the same gel, p-IRE1α and Vinculin correspond to the same gel and p-eiF2α and eiF2α correspond to the same gel. Each lane corresponds to a cell lysate from a pool of 80–100 islets isolated from one mouse. Similar results were obtained in pools of islets from 2 independent mice.

A previous study reported that olanzapine may activate UPR in beta cells through effects in proinsulin folding [16]. Therefore, we analyzed the effect of non-toxic concentrations of olanzapine in INS-1 beta cells on ER stress activation. Taking into account the viability assays, we chose olanzapine concentrations ranging from 1 to 6 µM for subsequent studies. As shown in Figure 2a, treatment of INS-1 cells with 1–6 μM olanzapine for a short time-period (4 h) increased the phosphorylation of the ER stress markers PERK, eIF2α and IRE1α. Likewise, olanzapine (6 μM) induced XBP1 splicing (Figure 2b). Olanzapine-induced phosphorylation of the PERK/eIF2α pathway was also evidenced in pancreatic islets from female mice (Figure 2c).

### 2.3. Twenty-Four Hour-Treatment with Non-Toxic Concentrations of Olanzapine Impaired Insulin Secretion in INS-1 Beta Cells

**Figure 3:**
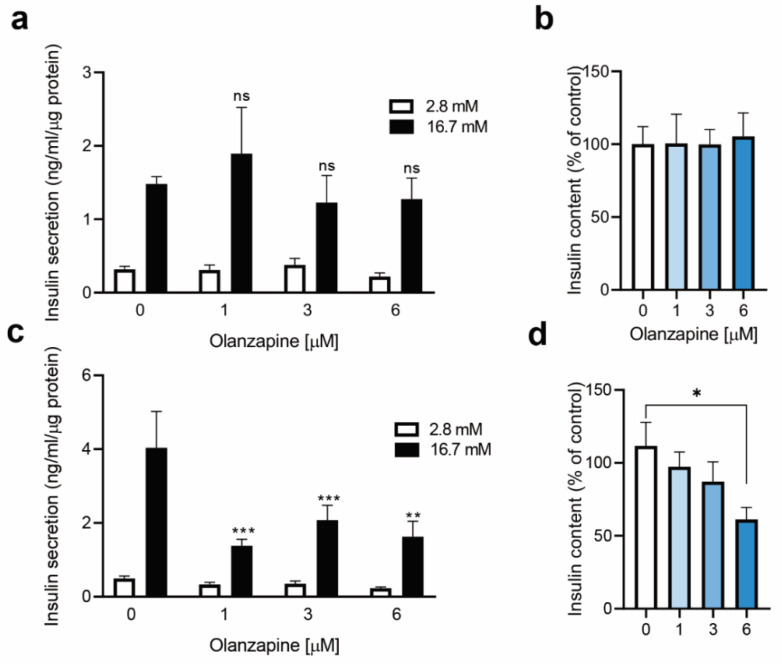
Effects of 4 or 24 h treatment with olanzapine on GSIS and insulin content in INS-1 beta cells. (**a**) GSIS in INS-1 beta cells upon olanzapine treatment for 4 h. (**b**) Insulin content at 4 h of olanzapine treatment. (**c**) GSIS in INS-1 beta cells upon olanzapine treatment for 24 h. (**d**) Insulin content at 24 h. Control: 0.01% DMSO was used as vehicle. Data are presented as means ± SEM of *n* = 3 independent experiments (3 replicates/condition), passage 30–36. In (**a**,**c**), *p*-values were analyzed by two-way ANOVA and Bonferroni post-hoc test. ns (non-significant), ** *p* < 0.01 and *** *p* < 0.001 versus control cells with 16.7 mM glucose. In (**d**), *p*-values were determined by one-way ANOVA and Bonferroni Post-hoc test. * *p* < 0.05, versus control (non-treated) cells.

As olanzapine triggered ER-stress-mediated signaling upon 4 h incubation in INS-1 cells (Figure 2a), we analyzed the possibility of non-toxic concentrations (1–6 µM) of this SGA interfering with insulin secretion. As shown in Figure 3a,b, neither insulin secretion (1.48 ± 0.09 ng/mL/µg protein in controls) nor insulin content were found significantly altered by olanzapine after this short-term treatment, suggesting that the effect of olanzapine is likely mediated by long-term effects in the insulin secretion machinery of INS-1 beta cells. To test this, INS-1 beta cells were exposed to olanzapine for a longer time period (24 h). Notably, this treatment reduced GSIS by 50–65 % (*** *p* < 0.001) (Figure 3c). Moreover, the insulin content of INS-1 cells was found to be significantly decreased when olanzapine was used at 6 μM (* *p* < 0.05) (Figure 3d).

### 2.4. Co-Treatment of INS-1 Beta Cells or Pancreatic Islets with Olanzapine and the Bile Acid TUDCA Reduces ER Stress Signaling and Prevents the Impairment in Insulin Secretion

**Figure 4:**
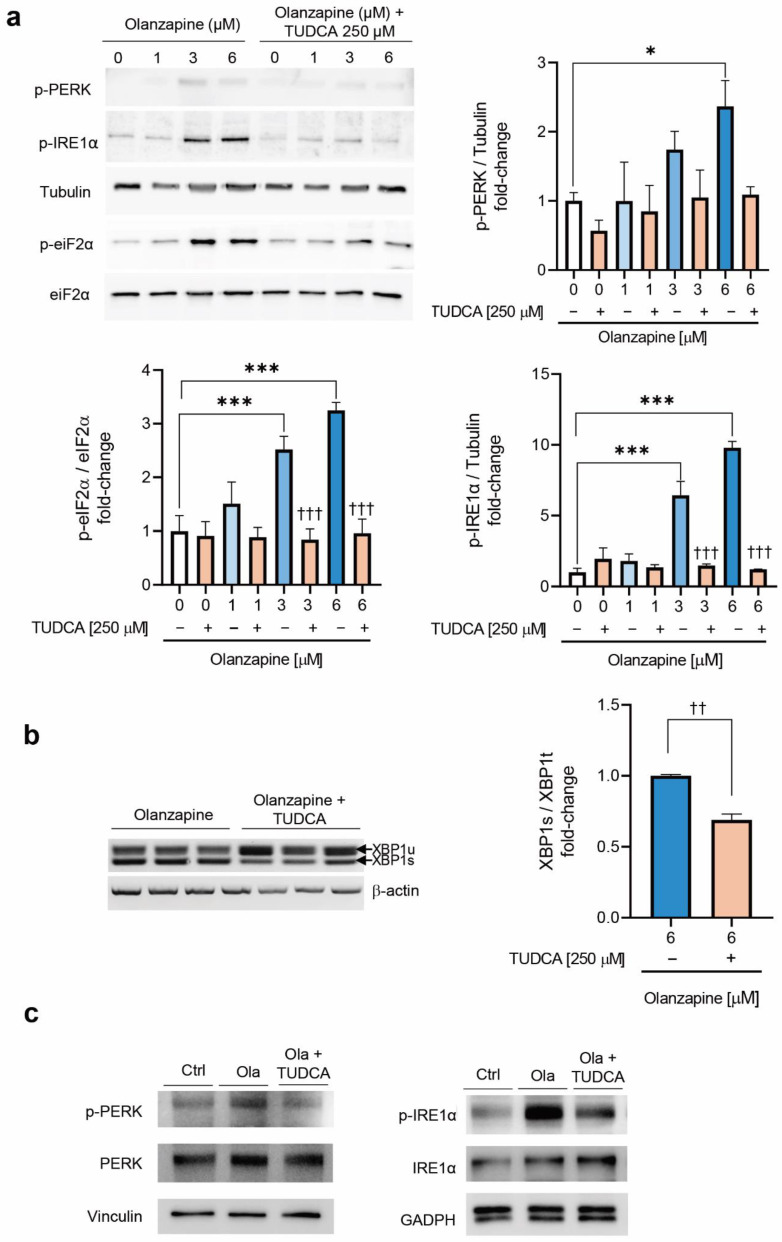
Alleviation of olanzapine-induced ER-stress-mediated signaling by TUDCA in INS-1 cells and pancreatic islets. (**a**) PERK, IRE1α and eIF2α phosphorylation in INS-1 beta cells upon co-treatment with olanzapine and 250 μM TUDCA. Control: 0.01% DMSO was added as vehicle. Quantification and statistical analysis of p-PERK, p-eIF2α and p-IRE1α levels. Representative Western blot images are shown. P-PERK, p-IRE1α and tubulin correspond to the same gel, and p-eiF2α and eiF2α correspond to the same gel. The graphs show the fold increase versus control cells. Tubulin and total eIF2α were used as loading controls. The experiments were repeated six times, independently, with INS-1 cells in different passages, with triplicate conditions in each one. Data are presented as mean ± SEM of *n* = 3 independent experiments (3 replicates/condition), passage 25–35. *p*-values were determined by one-way ANOVA and Bonferroni post-hoc test * *p* < 0.05, *** *p* < 0.001 compared to INS-1 treated with DMSO; ^†††^ *p* < 0.001 compared to co-treatment with the same dose of olanzapine and TUDCA. (**b**) XBP1 splicing in INS-1 beta cells upon co-treatment with 6 μM olanzapine and 250 μM TUDCA for 3 h. Data are presented as mean ± SEM of *n* = 2 independent experiments (3 replicates/condition). Differences between 2 groups were compared using Mann–Whitney U test: ^††^ *p* < 0.01 compared with olanzapine plus TUDCA. (**c**) Pancreatic islets from female mice were incubated with 6 μM olanzapine and 250 μM TUDCA for 4 h, and phosphorylation levels of PERK and IRE1α were analyzed by Western blot. Representative Western blot images from 2 independent experiments are shown. P-PERK, PERK and vinculin correspond to the same gel; p-IRE1α and IRE1 and GAPDH correspond to the same gel; and p-eiF2α and eiF2α correspond to the same gel. Each lane corresponds to a cell lysate from a pool of 80–100 islets isolated from one mouse. Similar results were obtained in pools of islets from 2 independent experiments.

Thereafter, we wondered if alleviation of ER stress with the bile acid TUDCA, which has been shown to reduce ER stress in various cell types, including beta cells [18], could counteract the reduction in insulin secretion by olanzapine in INS-1 beta cells and pancreatic islets. To achieve this, INS-1 beta cells were exposed to olanzapine (1–6 μM), in the absence or presence of TUDCA (250 μM), for 4 h. As shown in Figure 4a, co-treatment with TUDCA reduced olanzapine-mediated effects in the phosphorylation levels of PERK, IRE1α (^††^ *p* < 0.01, ^†††^ *p* < 0.001 vs control cells) and eIF2α (^†††^ *p* < 0.001 vs control cells) in INS-1 beta cells. Likewise, the combination of olanzapine (6 μM) and TUDCA counteracted XBP1 splicing (Figure 4b). The reductions in olanzapine-induced PERK and IRE1α phosphorylation were also observed in pancreatic islets from female mice (Figure 4c).

**Figure 5:**
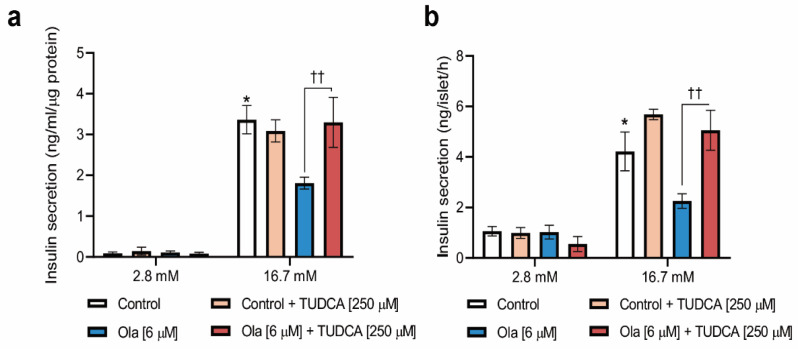
Restoration of insulin secretion in INS-1 cells and pancreatic islets by co-treatment with olanzapine and TUDCA. (**a**) GSIS in INS-1 beta cells co-treated with olanzapine (6 μM) in the absence or presence of TUDCA (250 μM) for 24 h. Data are presented as mean ± SEM of *n* = 3 independent experiments (3 replicates/condition), passage 25–35. (**b**) GSIS in pancreatic islets under the same experimental conditions described in (**a**). Experiments were performed in 3–4 replicates per condition in islets from *n* = 6 mice. *p*-values were determined by two-way ANOVA and Bonferroni post-hoc test. * *p* < 0.05, compared to INS-1 or islets treated with DMSO and stimulated with 16.7 mM glucose. ^††^ *p* < 0.01, compared to INS-1 cells or islets treated with olanzapine plus TUDCA and stimulated with 16.7 mM glucose.

Finally, we examined insulin secretion in INS-1 cells co-treated with olanzapine and TUDCA for 24 h. As shown in Figure 5a, TUDCA did not significantly change basal (2.8 mM) insulin secretion, either alone or in combination with olanzapine. Importantly, under conditions of 16.7 mM glucose, the combination of olanzapine and TUDCA maintained GSIS at similar levels to that of non-treated cells (Figure 5a), and both effects were further confirmed in pancreatic islets (Figure 5b). Altogether, our results strongly suggest that olanzapine can directly target pancreatic beta cells by activating ER-stress-mediated signaling, and alleviation of this effect can restore their functionality regarding insulin secretion.

## 3. Discussion

Increased incidence of T2D in patients under pharmacological treatment, a condition known as drug-induced T2D, has been reported in the last few years [19]. In the case of patients with schizophrenia receiving chronic treatment with SGAs, the risk of developing T2D can be four-fold higher compared to that of the healthy population [20,21]. In relation, metabolic disturbances associated with SGAs have been accurately modeled in preclinical studies, where pancreatic islets expressed a broad range of receptors targeted by SGAs, and therefore are susceptible of being altered by chronic treatment with these drugs [6].

We have recently reported that treatment of female mice with the SGA aripiprazole supplemented in the diet increased tryptophan hydroxylase 1 (TPH1) expression and serotonin production in pancreatic beta cells, causing beta cell dysfunction by interfering with the regulation of the serotoninergic system and also by inhibiting Ca^2+^ entry [22]. However, using a similar administration route, olanzapine did not alter the expression of relevant genes related to insulin synthesis or islet function. However, chronic treatment with olanzapine induced islet hyperplasia and hyperinsulinemia, and also inhibited insulin secretion in pancreatic islets from female mice without interfering with serotonin metabolism or Ca^2+^ signaling in beta cells. In this regard, a previous study conducted in rats receiving a single dose of olanzapine reported parallel decreases of insulin and c-peptide in circulation [23].

Interestingly, in our recent study we found that inhibition of insulin secretion in female mice receiving a chronic treatment with olanzapine seemed to be, at least in part, due to defective insulin granule maturation [22]. Amorphic insulin granules with reduced immunogold staining for mature insulin can reflect deficiencies in proinsulin processing at the ER, in which subsequent accumulation of misfolded proinsulin can exceed its functional capacity, thereby reducing insulin secretion [24]. For instance, Ninagawa et al. reported that olanzapine used at 10 µM inhibited proinsulin folding in mouse islets and the MIN-6 beta cell line by inducing aberrant disulfide bond formation, reducing insulin secretion [16]. Importantly, ER stress in beta cells has been described in both T1D [25] and T2D [26], and specific mutations in ER stress-related proteins have been linked to monogenic and syndromic diabetes mellitus [27]. In the context of T2D, it is widely accepted that pancreatic beta cells are exposed to a marked ER stress resulting from the increased insulin demand caused by hyperglycemia and obesity [28]. This has been evidenced in pancreatic islets from *db/db* mice in parallel to the alterations in glucose homeostasis [29].

Herein, we show activation of ER-stress-mediated signaling in INS-1 beta cells and pancreatic islets by olanzapine, particularly PERK/eIF2α and IRE1α phosphorylation, and XBP1 splicing, in line with the results reported by Ozasa et al. [11]. Importantly, our study was conducted using concentrations of olanzapine that preserved the viability of INS-1 beta cells and the morphology of pancreatic islets, thereby excluding off-target effects of this SGA on insulin secretion related with INS-1 cell death, an effect that has been attributed to eIF2α activation [30]. It is noteworthy to mention that in other studies, activation of the PERK/eIF2α pathway was found in the hypothalamuses of female rats treated with olanzapine [14], and in the prefrontal cortexes [15]. Our findings herein show that olanzapine directly targets beta cells by inducing ER stress, an effect that is likely responsible for subsequent alterations in beta cell functionality. Of relevance, olanzapine inhibited GSIS in INS-1 beta cells after long-term (24 h) incubation, but it did not elicit any effect in the short term (4 h). Therefore, inhibition of insulin secretion by olanzapine could rely on newly synthetized mediators upon activation of ER-stress-mediated signaling pathways [31]. It is noteworthy to mention that ER-stress-independent effects of olanzapine might also be involved in the inhibition of GSIS, since 1 µM olanzapine did not significantly increase the early ER-stress-mediated signaling (i.e., PERK, IRE1α and eiF2α phosphorylation), but it decreased GSIS after 24 h of incubation. Thus, it is tempting to propose that olanzapine receptors (i.e., dopamine, serotonin, muscarin and histamine) which are expressed on pancreatic beta cells [32], and/or their downstream signaling mediators, might also interfere with the insulin secretory machinery. This important issue deserves future research. Nevertheless, the specific contribution of ER stress to olanzapine-mediated beta cell dysfunction was demonstrated herein. Our results in INS-1 cells and pancreatic islets evidenced that co-treatment of INS-1 cells with olanzapine and TUDCA, a bile acid that attenuates UPR and prevents the subsequent ER stress in beta cell models [33,34], decreased eIF2α and IRE1α phosphorylation and XBP1 splicing in response to olanzapine, and importantly, prevented the reduction in GSIS in both INS-1 cells and islets. Since the deleterious effect of olanzapine on insulin secretion was reverted by the co-treatment with an ER stress inhibitor, individuals with schizophrenia receiving treatment with this SGA could follow a combinatorial therapy aimed to reduce ER stress-induced beta cell dysfunction.

## 4. Materials and Methods

### 4.1. Reagents

Fetal bovine serum (FBS) and culture media RPMI-1640 containing 11.1 mM glucose were purchased from Sigma-Aldrich (St. Louis, MO, USA). RPMI-1640 without glucose, L-glutamine, sodium pyruvate and penicillin/streptomycin were purchased from Gibco (Thermo Fisher Scientific, Waltham, MA, USA). Olanzapine was obtained from Glentham Life Sciences (UK) and stocked at 100 mM in DMSO at −20 °C. Tauroursodeoxycholic acid (TUDCA) was purchased from Sigma-Aldrich (T6260; TUDCA). All other chemicals were purchased form Sigma-Aldrich unless otherwise noted.

### 4.2. Culture of INS-1 (821/13) Beta Cell Line

The rat insulinoma INS-1 832/13 cell line (referred as INS-1) was kindly provided by Dr. Hindrik Mulder (Lund University Diabetes Centre, Lund, Sweden) [35]. Mycoplasma-tested INS-1 cells were maintained in RPMI-1640 containing 11.1 mM glucose and supplemented with 2 mM L-glutamine, 1 mM sodium pyruvate, 50 μM β-mercaptoethanol, 10 mM HEPES and 10% FBS in a humidified atmosphere of 37 °C and 5% CO_2_. Cells were passaged twice a week by trypsin incubation. INS-1 cells were seeded in 6-well tissue plates at a density of 6 × 10^5^ cells/well, and after 24 h cells were treated with olanzapine at concentrations of 1–6 μM, 250 μM TUDCA or vehicle (0.01% DMSO) for 3–4 or 24 h. For all compounds prepared in DMSO, the final concentration of this solvent in the culture medium was kept at less than 0.01%.

### 4.3. Cell Viability with Crystal Violet and MTT in INS-1 Cells

INS-1 cells were seeded at a density of 3 × 10^5^ cells/well in a 12-well plate, and the day after treated with some concentration (1–100 μM) of olanzapine. For assessment of cell viability using crystal violet, individual wells were fixed with 4% paraformaldehyde (PFA) for 15 min and washed with PBS. After, wells were incubated with a 0.1% crystal violet solution (HT901; Sigma-Aldrich, USA) for 20 min. Then, wells were washed with water and air dried for several days. A solution of 10% acetic acid was used to dissolve the crystal violet and absorbance was measured in this supernatant at 590 nm. The MTT (3-(4,5-dimethylthiazol-2-yl)-2,5-diphenyltetrazolium bromide) tetrazolium reduction assay was also used to measure cell viability (M6494; Thermo Fisher Scientific, USA). After 24 h of treatment, 50 µL of MTT solution (5 mg/mL) was added to each well and the plate was incubated at 37 °C for 3 h. Then, 150 µL of MTT solvent was added to the wells and the plate was incubated for 15 min. Absorbance was read at 590 nm.

### 4.4. GSIS Measurement in INS-1 Cells

INS-1 cells were seeded in 6-well plates, and when 90% confluence was reached, they were treated for 24 h with olanzapine at 1–6 μM. Krebs–Ringer buffer (KRB) solution containing (in mM) 2.8 glucose, 115 NaCl, 5 KCl, 1.2 NaHCO_3_, 1.1 MgCl_2_, 1.2 NaH_2_CO_4_, 2.5 CaCl_2_, 25 HEPES and 0.25% bovine serum albumin (BSA) was prepared with the addition of olanzapine with or without 250 μM TUDCA. Briefly, INS-1 cells were preincubated with 1 mL of KRB supplemented with 2.8 mM glucose for 30 min at 37 °C in an atmosphere of 5% CO_2_. Afterwards, the KRB solution was replaced with 1 mL of fresh KRB solution supplemented with 2.8 mM glucose, and INS-1 cells were then incubated for 1 h at 37 °C in an atmosphere of 5% CO_2_. The supernatant was collected into a 1.5 mL tube, and cells were incubated again with 1 mL of KRB solution supplemented with 16.7 mM glucose for 1 h at 37 °C in an atmosphere of 5% CO_2_. The supernatant was also collected into a 1.5 mL tube. Both tubes were centrifuged at 5600 rcf for 10 min at 4 °C, and the supernatants were collected and stored at −20 °C until analysis. Insulin was measured by ELISA (Mercodia, Uppsala, Sweden).

### 4.5. Analysis of Insulin Content in INS-1 Cells

After GSIS, 500 µL of glycine/NP-40 lysis buffer (200 mM glycine, 0.5% NP-40; pH 8.8) was added to the wells. INS-1 cells were scrapped, and the contents were transferred into a 1.5 mL tube. The samples were freeze–thawed in liquid nitrogen and a 37 °C water bath and then centrifuged for 15 min at 15,700 rcf. These supernatants were stored at −20 °C until analysis of insulin content. Values were normalized to protein content.

### 4.6. Isolation of Pancreatic Islets of Langerhans from Female Mice

Animal experiments were approved by the Animal Ethics Committees of the Spanish National Research Council and Comunidad de Madrid (reference PROEX 037/17, 2017) in accordance with Spanish (RD 53/2013) and European Union (63/2010/EU) legislation. Twelve-week-old female mice were sacrificed by cervical dislocation, and the pancreas was excised after infusing the animal in the common bile duct (CBD) with 3–4 mL of collagenase P solution (13.5 U/mL) derived from *Clostridium histolyticum* (11213865001; Roche, Mannheim, Germany) mixed with Hank’s balanced salt solution (HBSS) (1:14) (Sigma-Aldrich, USA). Islets were recovered in a 35 mm culture plate overnight at 37 °C and 5% CO_2_ in complete RPMI-1640 medium (2 mM L-glutamine, 1 mM sodium pyruvate, 50 μM β-mercaptoethanol, 10 mM HEPES and 10% (vol/vol) FBS) containing 5.6 mM glucose. The next day, islets were pooled, randomized and incubated with olanzapine at the indicated concentrations in the absence or presence of TUDCA (250 μM). Control islets were treated with 0.01% (vol/vol) DMSO.

### 4.7. Ex Vivo GSIS in Islets

Pancreatic islets, obtained as detailed above, were incubated in KRB containing (in mM) 115 NaCl, 5 KCl, 10 NaHCO_3_, 1.1 MgCl_2_, 1.2 NaH_2_CO_4_, 2.5 CaCl_2_, 25 HEPES, 2.8 glucose and 0.25% BSA, pH 7.4, for 1 h at 37 °C and 5% CO_2_. Thereafter, batches of 3 islets per condition matched by size were transferred into a 96-well plate containing 200 μL KRB per well with the addition of 2.8 and 16.7 mM glucose (final concentrations), and the plate was incubated at 37 °C and 5% CO_2_ for 1 h. After, 100 μL of the supernatant was collected and stored at −20 °C until the analysis. Experiments were performed with 4–6 replicates per condition and animal. Insulin concentration was determined with a Mouse Insulin ELISA kit following manufacturer’s instructions (10-1247-01; Mercodia, Uppsala, Sweden). Values were normalized per islet number.

### 4.8. Analysis of XBP-1 Splicing

Total RNA from INS-1 cells was extracted with Trizol^®^ reagent (15596026; Thermo Fisher Scientific, USA), and reverse transcription was performed using a High-Capacity cDNA Reverse Transcription Kit (436881; Thermo Fisher Scientific, USA) using random primers and Superscript III enzyme (18080093; Thermo Fisher Scientific; USA) according to the manufacturer’s instructions. RT-PCR was used for the detection of XBP1 mRNA splicing with forward (5′-CCTTGTAATTGAGAACCAGG-3′) and reverse (5′-CCAAAAGGATATCAGACTCGG-3′) primers as described [36].

### 4.9. Protein Extraction and Western Blotting

INS-1 cells were lysed in lysis buffer containing 10 mM Tris pH 7.5, 5 mM EDTA, 50 mM HCl, 30 μM sodium pyrophosphate, 50 mM NaF, 100 mM o-vanadate sodium, 1% Triton X-100, 1 mM phenylmethylsulfonyl fluoride (PMSF) and 10 µg/mL of protease inhibitors, pH 7.4–7.6. Pancreatic islets were lysed in Cell Lysis Buffer (#9803, Cell Signaling, Danvers, MA, USA) supplemented with 1 mM PMSF and 10 µg/mL of protease inhibitors. Protein extracts were quantified using the Bio-Rad Protein Assay solution (5000001, Bio-Rad, Hercules, CA, USA), and 15–30 µg was loaded in an 8–12% SDS polyacrylamide gel for electrophoresis (SDS-PAGE). Gels were transferred to PVDF membranes (IPVH00010; Merck). Then, membranes were incubated in blocking solution (5% skimmed milk in Tris-buffered saline pH 7.5 containing 0.05% Tween-20 (TBS-T) for 1 h at room temperature (RT), followed by o/n incubation (4 °C) with primary antibodies. Antibodies used were phospho-eIF2α (#9721, 1:1000, Cell Signaling, Danvers, MA, USA ), eIF2α (#9722, 1:1000, Cell Signaling), phospho-IRE1α (#NB100-2323, Novus Biologicals, UK), IRE1α (#3294, 1:2000 Cell Signaling), phospho-PERK (#3179, 1:1000, Cell Signaling), PERK (#5683, 1:1000, Cell Signaling), vinculin (sc-7314, 1:7500, Santa Cruz Biotechnology, Dallas, TX, USA), GAPDH (#G9545, 1:20,000, Sigma-Aldrich) and α-tubulin (#T5168, 1:20,000, Sigma-Aldrich). Blots were incubated with secondary anti-mouse (#sc-2005, 1:10,000, Santa Cruz Biotechnology) or anti-rabbit (#A120-108P, 1:25,000, Bethyl Laboratories Waltham, MA, USA) antibody. Membranes were developed with chemiluminescent substrate (Clarity Western ECL Substrate, 170-5060, Bio-Rad, Hercules, CA, USA), and different exposure times were used for each primary antibody with radiographic films in a radiology cassette (AGFA), or they were developed in a ChemiDoc imager (Bio-Rad). Densitometric analysis of the bands was performed using the Image J software (NIH, Bethesda, MA USA). Samples from each experiment were analyzed individually. Details of proteins detected in the same gel and the number of independent experiments and replicates are included in the corresponding figure legends.

### 4.10. Statistical Analysis

Statistical analysis was performed using GraphPad Prism software version 8 (Graph software, Inc, San Diego, CA, USA). A Shapiro–Wilk normality test was run to determine if the samples followed a parametric or non-parametric distribution. In parametric distributions, student’s T-test was used to compare mean differences between 2 groups, whereas one-way ANOVA with Bonferroni post-hoc test was used to compare mean differences between 3 or more groups. In non-parametric distributions, the Mann–Whitney U test was used to compare mean differences between 2 groups, and the Kruskal–Wallis test to compare mean differences between 3 or more groups. Two-way ANOVA was employed to compare 2 different categorical independent variables. Use of statistical analysis other than those described above is indicated in the figure legends. The data are presented as the mean ± SEM (standard error of the mean). Statistical significance was set to *, ^†^ *p* < 0.05, **, ^††^ *p* < 0.01, ***, ^†††^ *p* < 0.001.

## Data Availability

Data presented in this manuscript are available upon request from the corresponding author due to restrictions on privacy.
